# A concept map template to be used by medical students for displaying pathophysiological mechanisms within clinical cases

**DOI:** 10.15694/mep.2020.000039.1

**Published:** 2020-03-06

**Authors:** Marta Fonseca, Beatriz Oliveira, Pedro Carreiro-Martins, Nuno Neuparth, Antonio Rendas

**Affiliations:** 1 Nova Medical School; 2 Nova Medical School

**Keywords:** Concept map, medical education, meaningful learning, pathophysiology, template

## Abstract

This article was migrated. The article was marked as recommended.

**Background:** The use of concept maps (CMs) in health and medical education is increasing, particularly in the last decade. The research developed in this area has not yet clarified the role played by CMs in meaningful learning.

**Approach:** Our group developed a methodology, in a pathophysiology course, based on the classical CMs construction, using short clinical cases.

**Outcomes:** We propose a template that allows the display of the short clinical case embedded in the architecture of the CMs and connecting words targeted to specific pathophysiological mechanisms.

**Next Steps:** We consider that this experience can be extrapolated to the teaching and learning of pathophysiology in other health areas.

## Background

The use of concept maps (CMs) in health and medical education is increasing, particularly in the last decade ( Daley, Durning and Torre, 2016). The application of CMs to teaching and learning of diversified groups of students, mainly at the graduate level, is divided into two different approaches. In the first one, the starting point is the presentation of basic science concepts and its application to the understanding of the normal function or of the mechanisms of disease, followed by the construction of CMs to show the understanding of interrelated concepts ( González *et al*., 2008). In the second one, the use of data from healthy humans or patient information, either in the Problem-Based Learning (PBL) format or as short clinical cases (vignettes), is used as a trigger point to the study of the functional and pathological mechanisms with the CMs representing, visually, the overall acquired knowledge ( Veronese *et al*., 2013).

The complexity of the interrelated concepts related to learning health sciences and medicine is very different from the complexity of abstract scientific concepts used by Novak in his pioneer work on the development of CMs ( Novak, 2003).

From our perspective, this difference has created some difficulties in the construction of meaningful CMs in the context of health and medical education. Even taking into account the research developed in this area it is still not clear, from the literature, the role played by CMs in meaningful learning as applied to large groups of students, except in a PBL or a case-based format ( Krupat *et al*., 2016). In all the other cases, the visual representation of the knowledge acquisition applies only to the specific information, which is initially provided, for example, the relationship between stroke volume and cardiac output. The extrapolation of the acquired knowledge to a broader context is not always evident ( Burdo and Dwyer, 2015).

For this reason, the use of information from healthy people or patients is essential to provide this component of meaningful learning particularly for understanding the relations between basic sciences and normal or abnormal functional changes in a living human ( Vink *et al*., 2015). Due to the different techniques used to develop CMs, the direct application of Novak seminal work on scoring of CMs based on the “architecture” of the map may not be the most adequate method of assessment of meaningful learning in this context. In addition, attempts to score “a posteriori” the concepts that are more relevant may be affected by subjective criteria ( Torre, Durning and Daley, 2013).

Recently, attempts have made to reduce this difficulty using online concept maps with a standard format, designed by experts. However, this solution is mostly be applied to the final assessment of a large number of students and does not reflect the learning process that occurs, for example, during tutorial sessions or self-learning periods. Another online methodology used testable incomplete CMs, which proved to be successful to improve the learning of pathogenesis of renal and hepatic disease, but the study was performed in a small group of voluntary students. Very recently, a web-enabled mechanistic diagramming methodology was applied to two consecutive classes of 150 students, from a medical school with a fully integrated curriculum ( Ferguson *et al*., 2018). This approach used 16 mechanistic diagrams, developed by experts, and the students had to develop, individually, their own diagrams based on a clinical case and were allowed to use all the materials displayed in the reference map constructed by the expert. The students received one point for each correct matching between their maps and the expert maps in the identification of the relations between risk factors, etiology, pathogenesis and pathophysiology, and patients’ clinical findings. However, this approach was applied only to final assessments.

## Approach

Our group developed a similar methodology based on the classical CMs construction ( Rendas, Fonseca and Pinto, 2006), as described by Torre, with the additional inclusion of core concepts to be displayed and explained in CMs. These core concepts, previously identified by the staff, were communicated to the students. In addition to this communication, the students, working in small tutorial groups, received the patient information in the first session and proceed, in the following three sessions, to identify causes and mechanisms of the disease in relation to patients’ manifestations. The tutor, medically qualified, promoted the debate and assisted the students in the construction of the map using the freely available software program Cmap tools ^â^ (IHCM Cmaps Tools, Florida, USA). The final result was a map constructed with the collaboration of all the members of the tutorial group.

## Outcomes

**Figure 1.**
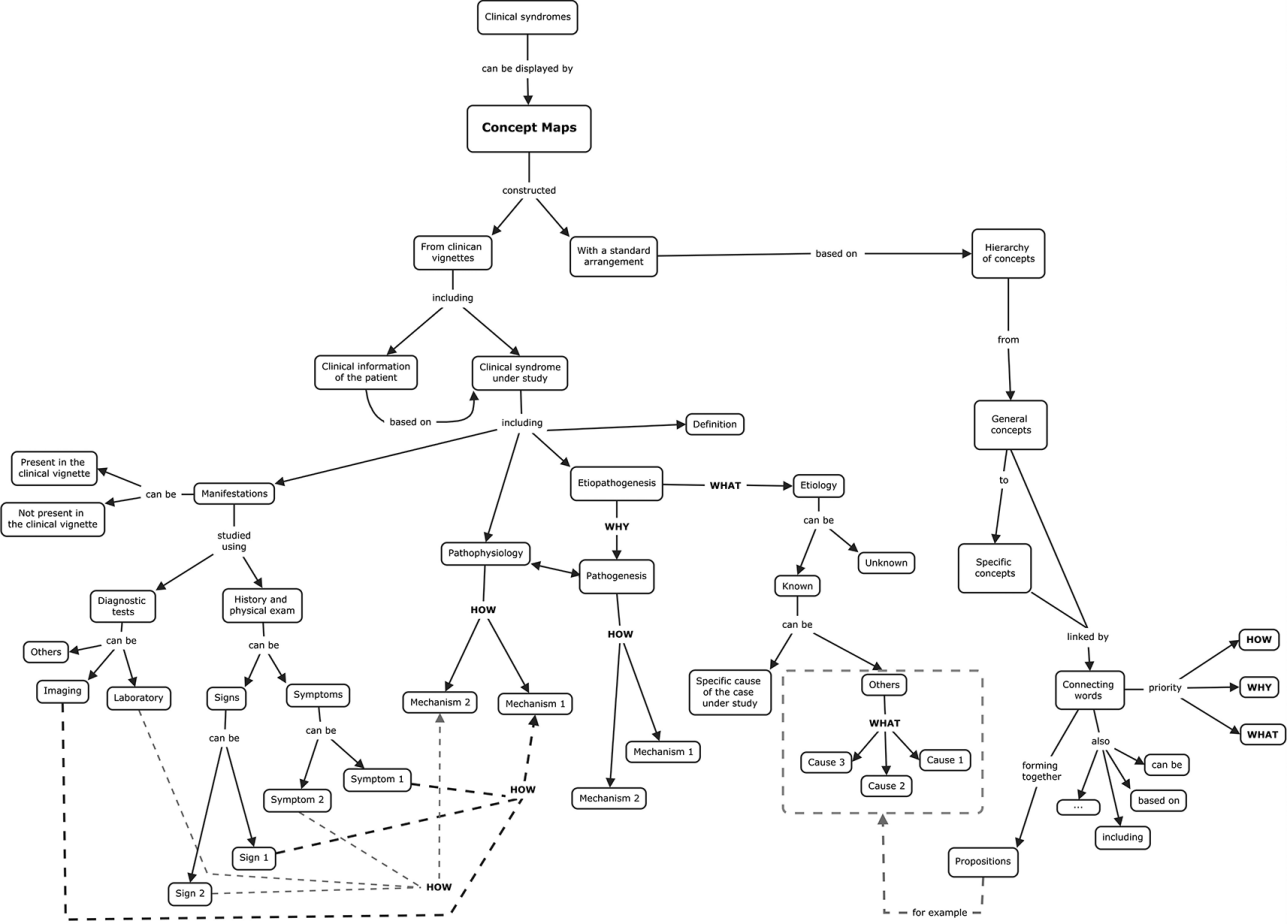
Template for the construction of concept maps in a pathophysiological course, allowing the integration of the clinical information in the architecture of the concept maps, using the connecting words HOW, WHY and WHAT.

Based on this experience, we propose a template ( Figure 1), to be given to the students in the first session at the same time as the clinical information and the core concepts. The template allows for the display of the clinical case embedded in the architecture of the CMs and the connecting words are targeted to specific mechanisms: “how” - pathophysiological; “why” - pathogenic; “what” - etiologic. This orientation does not exclude the use of other connecting words such as: “based on”, “can be”, “including”, and so forth. We also admit that establishing a hierarchy of concepts, from top to bottom, is important for the display of meaningful learning in this context. The same importance is given to cross-links between interrelated concepts. On the other hand, less importance is given to propositions. This template, in a more simplified version, has already been used with success, in our pathophysiology course particularly for the integration of basic science knowledge to patient’s manifestations ( Rendas, Fonseca and Pinto, 2006).

## Next Steps

We consider that this experience can be extrapolated to the teaching and learning of pathophysiology in other health areas.

## Take Home Messages

• The template allows for the display of the clinical case embedded in the architecture of the CMs in the context of teaching and learning pathophysiology to medical students.
• The connecting words of the CMs identify specific mechanisms: “how” - pathophysiological; “why” - pathogenic; “what” - etiologic.

## Notes On Contributors

Marta Fonseca, is Assistant of Pathophysiology at NOVA Medical School and has been teaching Pathophysiology to medical students using problem-based learning and concept maps. Is a PhD student at NOVA Medical School and is a practicing Family Physician. ORCID: https://orcid.org/0000-0001-5167-0598

Beatriz Oliveira, 5th year medical student at NOVA Medical School (NMS). Has been collaborating with the Pathophysiology Department of NMS regarding application of Concepts Maps in medical learning since 2017.

Pedro Carreiro-Martins, is Assistant Professor of Pathophysiology at NOVA Medical School and consultant of Allergy and Clinical Immunology. In the last 15 years has teached Pathophysiology to medical students using problem-based learning and concept maps. ORCID: https://orcid.org/0000-0002-4129-133X (h-index:14).

Nuno Neuparth, is Associate Professor of Pathophysiology, Head of the Department at NOVA Medical School and has an h-index 11. He is a clinician specialized in immunoallergology with a main interest in respiratory pathophysiology. He has been teaching pathophysiology to medical students using problem-based learning and concept maps for the last 40 years. ORCID: https://orcid.org/0000-0001-5149-7473

António Rendas, is Emeritus Professor of Pathophysiology. After his PhD in Experimental Pathology, University of London, Cardiothoracic Institute, Brompton Hospital, he founded the Department of Pathophysiology at NOVA Medical School, NOVA University Lisbon, committed to teaching and learning methods having introduced problem-based learning and concept mapping in an undergraduate medical curriculum in Portugal. This work has been published in international specialized journals. ORCID: https://orcid.org/0000-0001-5173-4256

## References

[ref1] Burdo J. and O’Dwyer L. (2015) The effectiveness of concept mapping and retrieval practice as learning strategies in an undergraduate physiology course. Adv Physiol Educ. 39(4), pp.335–340. 10.1152/advan.00041.2015 26628657

[ref2] Daley B. J. Durning S. J. and Torre D. M. (2016) Using Concept Maps to Create Meaningful Learning in Medical Education. MedEdPublish. 5(1), pp.1–29. 10.15694/mep.2016.000019

[ref3] Ferguson K. J. Kreiter C. D. Haugen T. H. and Dee F. R. (2018) Web-Enabled Mechanistic Case Diagramming: A Novel Tool for Assessing Students’ Ability to Integrate Foundational and Clinical Sciences. Acad Med. 93(8), pp.1146–1149. 10.1097/ACM.0000000000002184 29465452

[ref4] González H. L. Palencia A. P. Umaña L. A. Galindo L. (2008) Mediated learning experience and concept maps: a pedagogical tool for achieving meaningful learning in medical physiology students. Adv Physiol Educ. 32(4), pp.312–316. 10.1152/advan.00021.2007 19047509

[ref5] Krupat E. Richards J. B. Sullivan A. M. Fleenor T. J. (2016) Assessing the Effectiveness of Case-Based Collaborative Learning via Randomized Controlled Trial. Acad Med. 91(5), pp.723–729. 10.1097/ACM.0000000000001004 26606719

[ref6] Novak J. D. (2003) The Promise of New Ideas and New Technology for Improving Teaching and Learning. Cell Biol Educ. 2(2), pp.122–132. 10.1187/cbe.02-11-0059 12888848 PMC162189

[ref7] Rendas A. B. Fonseca M. and Pinto P. R. (2006) Toward meaningful learning in undergraduate medical education using concept maps in a PBL pathophysiology course. Adv Physiol Educ. 30(1), pp.23–29. 10.1152/advan.00036.2005 16481605

[ref8] Torre D. M. Durning S. J. and Daley B. J. (2013) Twelve tips for teaching with concept maps in medical education. Med Teach. 35(3), pp.201–208. 10.3109/0142159X.2013.759644 23464896

[ref9] Veronese C. Richards J., B. Pernar L. Sullivan A. M. (2013) A randomized pilot study of the use of concept maps to enhance problem-based learning among first-year medical students. Med Teach. 35(9), pp.e1478–e1484. 10.3109/0142159X.2013.785628 23617466

[ref10] Vink S. C. Van Tartwijk J. Bolk J. and Verloop N. (2015) Integration of clinical and basic sciences in concept maps: a mixed-method study on teacher learning. BMC Med Educ. 15(1), pp.20. 10.1186/s12909-015-0299-0 25884319 PMC4365534

