# Supplementary material for: A concept map template to be used by medical students for displaying pathophysiological mechanisms within clinical cases
Source: MedEdPublish (2016). 2020 Mar 6;9:39. [Version 1] doi: 10.15694/mep.2020.000039.1 (PMC10697434; doi:10.15694/mep.2020.000039.1)
Supplement: Peer review history

# Reviewer report: Reviewer response for version 1

**DOI:** 10.21956/mep.20008.r30501

**Published:** 2020-05-20

**Authors:** Ken Masters

**Affiliations:** 1 Sultan Qaboos University

This review has been migrated. The reviewer awarded 3 stars out of 5 An interesting paper on a concept map template to be used by medical students for displaying pathophysiological mechanisms within clinical cases. Some issues:• In the introduction, it would be a good idea to explain, in one paragraph, perhaps with an example, what a concept map is, because, if the reader does not know that, then everything else (until the template is revealed) in the paper is a bit of a mystery.• Although the same rigour of evaluation as a research article is not required, it would be useful to see some form of evaluation. The authors do say that “This template, in a more simplified version…patient’s manifestations” and give a 2006 reference, so it would be useful to give just a little detail about that evaluation, even though it is rather old. If there has been any form of piloting or independent review, that would be useful.• With that, the comment in the Next Steps is probably a little premature, because it would probably not be wise to extrapolate the template to other areas until it had been evaluated in its current form and current area. Perhaps the Next Steps should rather be a more detailed evaluation of the template.Minor:• “attempts have made to reduce” should probably be “attempts have been made to reduce”I look forward to a Version 2 of this paper in which these issues are addressed.

Reviewer Expertise:

NA

No decision status is available

# Reviewer report: Reviewer response for version 1

**DOI:** 10.21956/mep.20008.r30500

**Published:** 2020-03-14

**Authors:** John Cookson

**Affiliations:** 1 University of Worcester

This review has been migrated. The reviewer awarded 4 stars out of 5 thank you; interesting concept, I would like to know how it works out in practice. Are students helped or constrained by having the framework? If there was a clinical problem to start it off then WHERE, WHEN and WHO would also be important

Reviewer Expertise:

NA

No decision status is available

# Reviewer report: Reviewer response for version 1

**DOI:** 10.21956/mep.20008.r30503

**Published:** 2020-03-08

**Authors:** Sarmishtha Ghosh

**Affiliations:** 1 International Medical University

This review has been migrated. The reviewer awarded 4 stars out of 5 This article deals with a diagrammatic approach to enhance student learning. After discussion in small groups, most of the time what remains obscure is the depth in which the students have understood the concepts or the mechanisms in order to be able to transfer the knowledge later in the workplace. Therefore, it is essential to check their individual perception. Concept mapping or mechanism mapping provides such opportunities.However, the authors can try to study by not supplying the students with a template but rather allowing them to use their creativity to reflect on their understanding.Overall the paper provides a good theoretical idea on how CM can be used in small group discussions.

Reviewer Expertise:

NA

No decision status is available

# Reviewer report: Reviewer response for version 1

**DOI:** 10.21956/mep.20008.r30502

**Published:** 2020-03-06

**Authors:** Margaret Dow

**Affiliations:** 1 Mayo Clinic

This review has been migrated. The reviewer awarded 4 stars out of 5 This is a great theoretical concept with a solid base in learning theory. I would love to see and example of this applied to physiology rather than just the raw template, but look forward to trying the idea out. Will watch for follow up data documenting successes.

Reviewer Expertise:

NA

No decision status is available
